# Positioning of enhanced monofocal intraocular lenses between conventional monofocal and extended depth of focus lenses: a scoping review

**DOI:** 10.1186/s12886-023-02844-1

**Published:** 2023-03-14

**Authors:** Joaquín Fernández, Carlos Rocha-de-Lossada, Francisco Zamorano-Martín, Marina Rodríguez-Calvo-de-Mora, Manuel Rodríguez-Vallejo

**Affiliations:** 1 Qvision. Ophthalmology Department, VITHAS Almería Hospital, 04120 Almería, Spain; 2 Ophthalmology Department, VITHAS Málaga. 29016, Málaga, Spain; 3 Hospital Regional Universitario de Málaga. Plaza del Hospital Civil, 29009 Málaga, S/N Spain; 4 Departamento de Cirugía, Área de Oftalmología. Doctor Fedriani, Universidad de Sevilla, 41009 Sevilla, S/N Spain; 5 Instituto de Oftalmología Conde de Valenciana, Ciudad de Mexico, 06800 México

**Keywords:** Intraocular lens, Cataract, Monofocal, Enhanced, Plus, Intermediate

## Abstract

**Background:**

New intraocular lenses (IOLs) have emerged since the originally coined monofocal and multifocal IOLs. The extended depth of focus (EDoF) and enhanced monofocal IOLs (mono-EDoF) that have appeared in the last decade have caused some confusion in their classification. The aim of this review was to summarize the outcomes provided by mono-EDOF IOLs and to determine which of the endpoints, described by the American National Standard (ANSI) for EDoF IOLs, are fulfilled.

**Methods:**

The MEDLINE, EMBASE, and WEB OF SCIENCE databases were searched. Two independent reviewers screened the studies for inclusion and data extraction. The search strategy was limited to studies published between 2020 and 2022, but not by language. The results are presented as a narrative summary accompanied by tables, in alignment with the objectives of this scoping review. Compliance with the endpoints for clinical outcomes described in the American National Standard Z80.35–2018 (ANSI) for EDoF lenses was checked and additional endpoints were defined.

**Results:**

Two systematic reviews, 13 laboratory, 21 clinical, and two mixed studies were included. Tecnis Eyhance was the mono-EDOF with the highest volume of evidence to date. Although laboratory studies included other IOLs, clinical evidence for them is still scarce, with only one study of IsoPure compared to a standard monofocal IOL. Evidence in comparison to EDoF lenses is also scarce, even for Tecnis Eyhance, with only three studies including this lens in comparison to an EDoF lens. After evaluation of the ANSI criteria, agreement was found in the failure for the increase in depth of field equal to or greater than 0.5 D for a visual acuity (VA) level of 0.2 logMAR and none of the studies supported that the median monocular VA at intermediate distance was at least 0.2 logMAR.

**Conclusions:**

Additional clinical evidence is required for other mono-EDOF IOLs beyond Tecnis Eyhance. Until the arrival of a standard classification, mono-EDOF should be better still classified as monofocal because the ANSI standards were not fully met.

**Supplementary Information:**

The online version contains supplementary material available at 10.1186/s12886-023-02844-1.

## Introduction

A monofocal intraocular lens (IOL) is a medical device implanted in the eye to restore distant vision in eyes with cataract. Beyond monofocal IOLs, other technologies such as simultaneous vision lenses (SVL) have also been developed to restore intermediate and/or near vision. SVL have been historically classified as multifocal IOLs, for which the light is split into multiple foci, and extended depth of focus lenses (EDoF), for which the far-distance focus is extended [1, 2].

A new technology named Tecnis Eyhance (Johnson & Johnson) was introduced in 2019 which as EDoF lenses extend the far distance focus [3]. Since this technology extends the depth of focus in a similar way to EDoF lenses, some confusion emerged among anterior segment surgeons, some declaring this lens as EDoF even though the IOL was launched as a new generation of monofocal intraocular lens. Subsequently, other IOLs with hypothetical similar visual performance were launched, and a new category popularly known as plus monofocal, mono-EDoF, or enhanced monofocals appeared. The new generation of monofocal IOLs that enhance intermediate vision might include Tecnis Eyhance (Johnson & Johnson), IsoPure (Physiol), Xact (Santen), Zoe (OphthalmoPro GmbH), RayOne EMV (Rayner), Lentis Quantum (Teleon Surgical), Evolux (Sifi), Vivinex Impress (Hoya), and Extend HP (Hanita Lenses).

Standards have not yet considered these lenses as a new category of SVL; therefore, they should still be classified as monofocal or EDoF lenses. The definition of EDoF has been reported by the American National Standard Z80.35–2018 (ANSI) [4], and describes up to four effectiveness end-points that should be met in full for classifying an IOL as EDoF, between them: (1) to demonstrate a statistical superiority over a control monofocal group on mean, monocular photopic distance-corrected intermediate visual acuity at 66 cm; (2) to demonstrate at least 0.5 D greater monocular photopic negative lens induced distance-corrected depth of focus compared to the monofocal control IOL at 0.2 logMAR visual acuity threshold; (3) the median, monocular distance-corrected photopic intermediate visual acuity at 66 cm is at least 0.2 logMAR, and (4) the mean, monocular photopic best corrected distance acuity for the EDoF IOL is statistically non-inferior to the control using a non-inferiority margin of 0.1 logMAR [4].

Unfortunately, the standard described by the ANSI requires comparison with a monofocal IOL in a randomized clinical trial, [4] and common published post-marketing studies are case series without a control lens. A case series without a control group does not allow for the evaluation of requirements 1, 2, and 4, as described by the ANSI. In addition, the descriptive statistic of the median described in the third requirement is not always reported since clinical studies usually offer a mean value. The inclusion of other absolute end-points instead of a relative comparison with a monofocal intraocular lens would help to describe the functional performance of the IOL from case series studies without a control group. Furthermore, these absolute values are expected to be included in future standard updates [5].

A preliminary search was conducted and two systematic reviews on the topic were identified [6, 7]. The purpose these reviews was to assess the efficacy, spectacle independence, patient satisfaction, and adverse event rates in comparison with monofocal IOLs. These systematic reviews concluded that new enhanced monofocal IOLs increased the intermediate and near vision in comparison to conventional monofocal IOLs, [6, 7] without compromising the contrast sensitivity or inducing photic phenomena [6]. These reviews required a narrow scope including only an intervention IOL (Tecnis Eyhance) in comparison of other monofocal IOLs, [6] or even a particular monofocal model [7]. Conversely, this is the first scoping review covering a wide scope that explores not only the clinical results of enhanced monofocal IOLs in comparison to standard monofocal IOLs, but also in comparison to EDoF lenses and laboratory studies. In the absence of a standard definition for these new lenses and to avoid confusion with EDoF lenses, the aim of this scoping review was to summarize the clinical and laboratory outcomes provided by enhanced monofocal IOLs (*Concept*) and to determine which of the endpoints, described by the ANSI for EDoF lenses and some additional endpoints that might be included in future standards (*Context*), are fulfilled in patients undergoing cataract surgery (*Population*).

### Review question

The following questions were addressed in this scoping review.

- What are the optical quality and profiles described in laboratory studies of enhanced monofocal lenses?
- What are the functional outcomes of patients implanted with enhanced monofocal IOLs for cataract surgery?
- Which efficacy endpoints, described by the standard (ANSI) and additional ones, are fulfilled for enhanced monofocals?

### Keywords

Enhanced monofocal, monofocal plus, mono-EDoF, EDoF, simultaneous vision.

### Eligibility criteria

#### Participants

This scoping review considered studies involving patients who underwent cataract surgery with bilateral implantation of an enhanced monofocal IOL in the capsular bag, and studies evaluating IOLs in the laboratory through the measurement of optical quality or optical profile. The following exclusion criteria were applied for the clinical studies: age < 19 years, comorbidities, history of corneal laser refractive surgery, postoperative complications as the main study purpose, eyes requiring toric IOL implantation or additional corneal incisions (astigmatism > 1.5 D), combining different targets (micro-monovision), or different IOL models (mix-and-match) between eyes.

#### Concept

The main purpose of the eligible clinical studies was to provide monocular and binocular postoperative clinical results considering the following outcomes: visual acuity at several distances, defocus curves, contrast sensitivity function, and patient-reported outcomes such as the percentage of patients achieving spectacle independence, satisfaction, positive photic phenomena perception, positive dysphotopsia, and recommendation or decision to undergo the same IOL implantation. Eligible laboratory studies reported the results of optical quality or surface profiles.

#### Context

At least one of the following IOLs was included in the study: Tecnis Eyhance (Johnson & Johnson), IsoPure (Physiol), Xact (Santen), Zoe (OphthalmoPro GmbH), RayOne EMV (Rayner), Lentis Quantum (Teleon Surgical), Evolux (Sifi), Vivinex Impress (Hoya), and Extend HP (Hanita Lenses).

#### Types of Sources

This scoping review considered original studies, either case series (CS) or randomized clinical trials (RCT), systematic reviews, meta-analyses, case reports, and comments. The sources of gray literature were excluded. No language restrictions were applied.

### Methods

The scoping review was conducted in accordance with the JBI methodology for scoping reviews, [8] and following the Preferred Reporting Items for Systematic Reviews and Meta-Analyses extension for Scoping Reviews (PRISMA-ScR) [9]. This review was conducted in accordance with the a priori protocol registered in the Open Science Framework (https://osf.io/m7wcu). A stakeholder and experts in IOLs were consulted when preparing the study protocol and when discussing the scoping review results as recommended by the JBI Manual for Evidence Synthesis [10]. The following deviations occurred from the a priori protocol: inclusion of the Extend HP (Hanita Lenses) in the Context, the addition of new key references found through snowballing techniques for manuscript published from the search date to the data extraction date starting on 3^th^ of November 2022, and a third independent reviewer was included to resolve discrepancies.

#### Search strategy

The search strategy and the initial and secondary searches were conducted by one of the reviewers using the 2Dsearch tool (UXLabs Limited) and checked for adequacy by a second reviewer. The search strategy aimed to locate only published studies. An initial limited search of MEDLINE (PubMed) and Google Scholar for the first 200 references was performed by using several keywords to identify relevant and irrelevant studies. A keyword search was performed to identify commonly used words in titles and abstracts, and the index terms assigned to these references were explored using PubReMiner (Jan Koster (AMC)). In the second stage, the scope of the search was optimized to maximize sensitivity such that all previously identified relevant references were retrieved, and the major possible number of irrelevant references was omitted. The text words contained in the titles and abstracts of the articles and the index terms used to describe the articles were used to develop a full search strategy for PubMed on October 2, 2022. The search strategy, including all the identified keywords and index terms, was adapted for EMBASE (Elsevier) and Web of Science (Isi Web of Knowledge). Specific year ranges were applied to retrieve studies on enhanced monofocal IOLs from 2020 to 2022. Language filtering was not applied. A detailed search strategy is presented in supplemental file A. New references published from the systematic search to the date of data extraction on November 3, 2022, were identified through snowballing search techniques, executing the search algorithm again, and looking for those published in the last month.

#### Study/source of evidence selection

Following the search, all identified citations were collated and uploaded to the Rayyan platform (Qatar Computing Research Institute, Doha, Qatar) for screening. Two independent reviewers screened titles and abstracts to assess the inclusion and exclusion criteria. The full text of the selected citations was assessed in detail against the inclusion criteria by the same two independent reviewers. The reasons for excluding sources of evidence at first and full-text screenings that did not meet the inclusion criteria were recorded and reported in this scoping review. Any disagreements between the reviewers at each stage of the selection process were resolved by a third independent reviewer.

#### Data extraction

Data were extracted from the papers included in the scoping review by two independent reviewers using a data extraction tool developed by the reviewers and described in the protocol. The extracted data included specific details about the participants, concept, context, study methods, and key findings relevant to the review questions. For studies that included plots for reporting results, as is habitual for defocus curves and contrast sensitivity, data were extracted from the images by one of the reviewers using the WebPlotDigitizer (Ankit Rohatgi) tool.

A new draft data extraction tool, not included in the protocol, was developed during the course of the review for laboratory studies because of the non-uniformity of the reported results for this concept. Any disagreements between the reviewers in data extraction were resolved by discussion. Missing data was filled as “Not Available” (NA), and no requests were made to authors for missing or additional data. Critical appraisal was not conducted, because it was not required for scoping reviews.

#### Data analysis and presentation

Tables and figures were used to present the data and illustrate the scoping review findings, as described in the protocol for clinical studies. A narrative summary describes how the results relate to the objectives and review questions. Different metrics were reported in the laboratory studies; therefore, the organization for presenting these data was decided during scoping review writing, instead of the protocol. The criteria for selecting the indices shown in the results section were the capability of the information to be summarized in single indices and the uniformity shown in the included manuscripts to describe common indices.

### Patient and public involvement

There was no patient or public involvement in this scoping review.

## Results

### Study inclusion

**Fig. 1:**
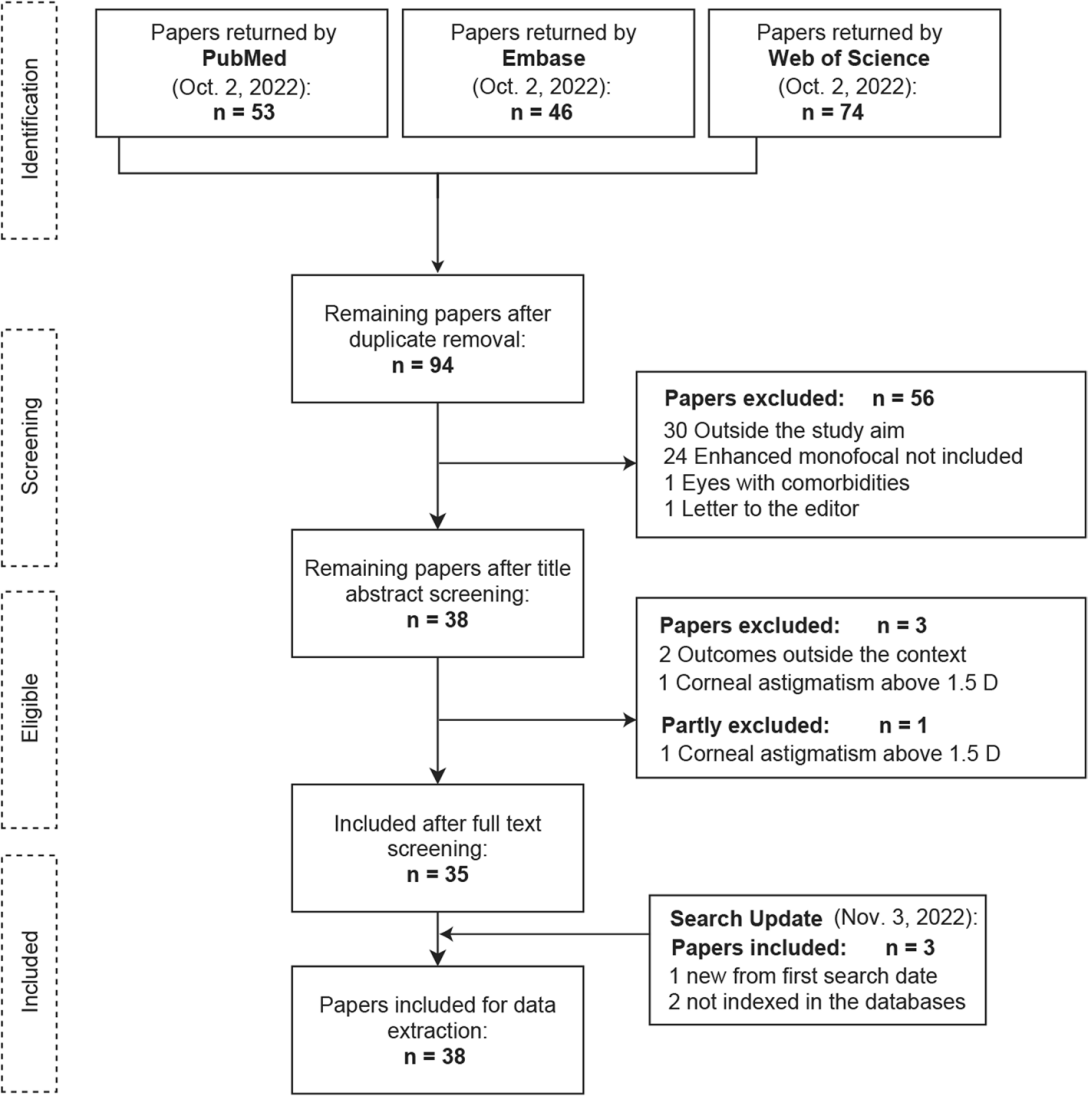
Flow diagram describing the number of sources of evidence screened, assessed for eligibility, and included in the review, with reasons for exclusion at each stage

Ninety-four papers were identified in the database search after duplicate removal and 56 were excluded after screening by title and abstract. Thirty of them were unrelated to the study aim and were obtained from a Web of Science database search, which had less specificity. Twenty-four were excluded because none of the IOLs described in the context were included, and only two papers were excluded because of comorbidities or different study types from those described in the inclusion criteria. After the full text evaluation, three clinical studies were excluded since the inclusion criteria was not accomplished, [11–13] and one mixed was maintained (partly excluded) since the criteria was accomplished in the laboratory study context but not in the clinical [14]. During the search update in the moment of data extraction, three papers were included, two not indexed in the databases and found through snowballing techniques, [15, 16] and 1 new after search update [17]. Finally, 38 studies were included in the data extraction process (Fig. 1).

### Characteristics of included studies

Two systematic reviews, 13 laboratory studies, 21 clinical studies (five RCT and two CS), and two mixed studies were included. For one of the mixed studies, only the laboratory data was extracted since the inclusion criteria was not accomplished for the clinical data [14].

### Laboratory studies

With regard optical quality metrics, the depth of focus achieved for predicted visual acuity of 0.2 logMAR and the increase range in comparison to a standard monofocal was retrieved for 4 manuscripts (Table 1) [15, 18–20]. Three papers showed the influence of the IOL centration on the modulation transfer function (MTF) at 50 lp/mm (Table 2) [21–23]. The wavefront spherical aberration was also reported in 3 studies (Table 3), [14, 19, 24] and the data provided by four manuscripts was not able to be compared since the MTF was normalized, single indices were not used or the data was presented for specific wavelengths or no common metrics [25–28]. Thus the findings of these studies are reported directly in the text as follows. Only one study reported the relative light intensity at 3 mm showing the Depth of Focus (DoF) achieved for an EDoF and three enhanced monofocal IOLs [27]. The major loss of energy at far distance was related to the increase of DoF in the following order: EDoF, Vivinex Impress, Xact, Tecnis Eyhance, and IsoPure [27]. These results were on agreement with other metrics such as the through focus MTF at 50 lp/mm which showed half MTF at far distance of the EDoF IOLs but a higher increase of the DoF in comparison to the RayOne EMV and the Tecnis Eyhance [25]. Only one study reported the chromatic performance of an enhanced monofocal, particularly the Xact [26]. The halo size was measured through the PSF-cross section showing similar profiles of enhanced monofocals Tecnis Eyhance, Zoe and IsoPure in comparison to the standard monofocal [18–20].

**Table 1:** Achieved depth of focus (DoF) range and increase (ΔDoF) in Diopters of an enhanced monofocal in comparison to a standard monofocal intraocular lens for predicted visual acuity (sVA) at 0.2 logMAR estimated from optical quality metrics

Author (Year)	Measurement device	Spherical aberration of cornea	Enhanced Monofocal	Predictor Metric	Aperture (mm)	DoF Range [ΔDoF]
Alarcon et al. (2020) [18]	Optical Bench	Unspecified + Z40	Tecnis Eyhance	sVA (0.2)	3	1.73 [0.5]
Vega et al. (2020) [19]	Optical Bench	+ 0.18 µm at 5 mm	Tecnis Eyhance	MTFa (11.4)^a^	3	1.89 [0.3]
Labuz et al. (2021) [20]	OptiSpheric	+ 0.28 µm at 5.15 mm	RayOne EMV	wOTF (0.51)^b^	3	1.49 [0.07]
Azor et al. (2022) [15]	Optical Bench	+ 0.18 µm at 5 mm	Tecnis Eyhance	MTFa (11.4)^a^	3	1.83 [0.3]
Azor et al. (2022) [15]	Optical Bench	+ 0.18 µm at 5 mm	IsoPure	MTFa (11.4)^a^	3	1.41 [0]
Labuz et al. (2021) [20]	OptiSpheric	+ 0.28 µm at 5.15 mm	IsoPure	wOTF (0.51)^b^	3	1.70 [0.28]
Labuz et al. (2021) [20]	OptiSpheric	+ 0.28 µm at 5.15 mm	ZOE	wOTF (0.51)^b^	3	2.07 [0.65]
Labuz et al. (2021) [20]	OptiSpheric	+ 0.28 µm at 5.15 mm	Tecnis Eyhance	wOTF (0.51)^b^	3	2.25 [0.82]

MTFa Area under the modulation transfer function, wOTF Weighted optical transfer function

^a^Equivalent to 0.2 logMAR according to Vega et al. [29]

^b^Equivalent to 0.2 logMAR according to Alarcon et al. [30]

**Table 2:** Modulation transfer function at 50 line pairs per millimeter (MTF 50lp/mm) for enhanced monofocal intraocular lenses tested at two different apertures and under three testing conditions

Author (Year)	Measurement device	Spherical aberration of cornea	Enhanced Monofocal	Conditions	Aperture (mm)	MTF 50lp/mm
Borkenstein et al. [21]	OptiSpheric	+ 0.28 µm at 5.15 mm	Acunex Quantum	Centered	3 / 4.5	0.61 / 0.40
Borkenstein et al. [21]	OptiSpheric	+ 0.28 µm at 5.15 mm	Acunex Quantum	1 mm Decentered	3 / 4.5	0.41 / 0.26
Borkenstein et al. [21]	OptiSpheric	+ 0.28 µm at 5.15 mm	Acunex Quantum	5° Tilted	3 / 4.5	0.55 / 0.34
Borkenstein et al. [22]	OptiSpheric	+ 0.28 µm at 5.15 mm	Lentis Quantum	Centered	3 / 4.5	0.61 / 0.33
Borkenstein et al. [22]	OptiSpheric	+ 0.28 µm at 5.15 mm	Lentis Quantum	1 mm Decentered	3 / 4.5	0.49 / 0.26
Borkenstein et al. [22]	OptiSpheric	+ 0.28 µm at 5.15 mm	Lentis Quantum	5° Tilted	3 / 4.5	0.58 / 0.35
Schmid et al. [23]	OptiSpheric	+ 0.28 µm at 5.15 mm	Tecnis Eyhance	Centered	3 / 4.5	0.61 / 0.44
Schmid et al. [23]	OptiSpheric	+ 0.28 µm at 5.15 mm	Tecnis Eyhance	1 mm Decentered	3 / 4.5	0.40 / 0.18
Schmid et al. [23]	OptiSpheric	+ 0.28 µm at 5.15 mm	Tecnis Eyhance	5° Tilted	3 / 4.5	0.38 / 0.15
Schmid et al. [23]	OptiSpheric	+ 0.28 µm at 5.15 mm	RayOne EMV	Centered	3 / 4.5	0.41 / 0.22
Schmid et al. [23]	OptiSpheric	+ 0.28 µm at 5.15 mm	RayOne EMV	1 mm Decentered	3 / 4.5	0.42 / 0.18
Schmid et al. [23]	OptiSpheric	+ 0.28 µm at 5.15 mm	RayOne EMV	5° Tilted	3 / 4.5	0.40 / 0.18

**Table 3:** Wavefront spherical aberration for coefficients Z40 and/or Z60 and reported apertures

Author (Year)	Technique	Measurement device	Enhanced Monofocal	Coefficient	Aperture (mm)	Endpoint
Schmid et al. [24]	Shack-Hartmann	WaveMaster IOL 2	Tecnis Eyhance	Z40 / Z60 (µm)	5.8	-0.93 / 0.02
Schmid et al. [24]	Shack-Hartmann	WaveMaster IOL 2	RayOne EMV	Z40 / Z60 (µm)	5.8	0.27 / -0.12
Vega et al. (2020) [19]	Shack-Hartmann	HASO 76	Tecnis Eyhance	Z40 (µm)	3 / 4.5	-0.07 / -0.25
Fernández-V-C et al. [14]	Phase-shifting	NIMO TR1504	Tecnis Eyhance	Z40 (µm)	3 /4.5	0.07 / -0.20

With regard to surface profiles, two manuscripts presented the profile of the Tecnis Eyhance measured with the Talysurf CLI 1000 and Zeta Z300 profilometers and in comparison to standard monofocal and refractive EDOF lenses [31, 32]. These studies demonstrated variations between the maximum and minimum elevations of the profiles below ± 1 µm for the standard monofocal, ± 2 µm for the enhanced monofocal, and ± 4 µm for the refractive EDoF.

### Clinical studies

Three clinical studies compared enhanced monofocals with EDoF lenses, [33–35] 14 compared the clinical results with a standard monofocal, [3, 17, 28, 36–46] and three case series without a comparison group were identified [16, 47, 48] Only one study included both monofocal and EDoF lenses in comparison to Tecnis Eyhance [49]. Supplemental file B contains all the data extracted from clinical studies.

Table 4 shows the endpoints described in each of the enhanced monofocal studies, and the Supplemental A file contains all data extracted from these studies. All the studies comparing EDoF lenses (Symfony, Eyecril SERT and Vivity) with enhanced monofocal lenses were conducted with the Tecnis Eyhance [33–35, 49]. General agreement was reported about a higher depth of focus range for EDOF lenses and statistically significant differences only at near vision [33–35, 49]. Despite of the higher range of the depth of focus showed in the defocus curve and the statistically significant differences at near, no significant differences were reported for visual acuities at intermediate vision [33–35]. The comparison with the standard monofocals is presented in the next section.

**Table 4:** Clinical results that have been described in enhanced monofocal studies

**Author (Year)**	**Year**	**Study type**	**Mono-EDOF**	**Compared with**	**Efficacy **	**DC**	**CS**	**SI**	**Satisfaction**	**PP**	**PD**	**Recom.**
Bova et al [44]	2022	CS	IsoPure	PCB00	M&B,U&C,D&I	M&B	B,P,WG	No	No	No	No	No
Nanavaty et al [39]	2022	RCT	Tecnis Eyhance	RayOne	M&B,U&C,D&I	M&B	No	No	No	Yes	No	No
Corbelli et al [49]	2022	CS	Tecnis Eyhance	2 lenses	M&B,U&C,D&I&N	B	B,P,WG	Yes	No	No	No	No
Lopes et al [40]	2022	CS	Tecnis Eyhance	PCB00	M&B,U,D&I	B	No	No	Yes	Yes	No	No
Steinmüller et al [17]	2022	CS	Tecnis Eyhance	ZCB00	M&B,U&C,D&I&N	M&B	All-M,Me	No	No	Yes	No	No
Sabur et al [33]	2022	CS	Tecnis Eyhance	Vivity	M&B,U&C,D&I&N	B	No	Yes	Yes	No	Yes	No
Lee et al [34]	2022	RCT	Tecnis Eyhance	Symfony	M&B,U,D&I&N	M&B	No	Yes	Yes	Yes	Yes	Yes
Auffarth et al [45]	2021	RCT	Tecnis Eyhance	ZCB00	M&B,U&C,D&I	B	M,P,Me,G,WG	No	No	No	Yes	No
Ucar et al [37]	2021	CS	Tecnis Eyhance	ZCB00	M,U&C,D&I&N	M	No	No	No	No	No	No
Kang et al [41]	2021	CS	Tecnis Eyhance	ZCB00	M,U,D&I&N	M	No	No	No	No	No	No
Unsal et al [38]	2021	CS	Tecnis Eyhance	ZCB00	M&B,U&C,D&I	B	No	Yes	No	No	Yes	No
Cinar et al [42]	2021	CS	Tecnis Eyhance	SN60WF	M,U&C,D&I&N	No	No	Yes	No	No	No	No
Huh et al [28]	2021	CS	Tecnis Eyhance	ZCB00	B,U&C,D&I&N	B	No	No	No	Yes	No	No
Jeon et al [35]	2021	CS	Tecnis Eyhance	Symfony	M,U,D&I&N	M	No	No	No	No	No	No
Baur et al [47]	2021	CS	xact ME4	None	M&B,U&C,D&I	M&B	All	No	No	No	No	No
Tañá-Sanz et al [48]	2021	CS	xact ME4	None	Not	M	No	No	No	No	No	No
Stodulka et al [16]	2021	CS	IsoPure	None	M&B,C&U,D&I	M&B	All	No	No	No	No	No
Yangzes et al [36]	2020	CS	Tecnis Eyhance	ZCB00	M,U,D&I&N	M	No	No	No	No	No	No
Eguileor et al [43]	2020	RCT	Tecnis Eyhance	ZCB00	M&B,C,D&I	B	No	No	No	No	No	No
Mencucci et al [3]	2020	CS	Tecnis Eyhance	ZCB00	M&B,U&C,D&I&N	B	B,P,WG	Yes	No	No	No	No
Garzón et al [46]	2022	RCT	Tecnis Eyhance	ZCB00	M,U&C,D	No	M,P,WG	No	No	No	No	No

*Mono-EDOF* Enhanced monofocal, *DC* Defocus curve, *CS* Contrast Sensitivity, *SI* Spectacle independence, *PP* Photic phenomena, *PD* Positive dysphotopsia, Recom: Recommended to familiars of being operated again, Results reported beyond Yes or No: *M* Monocular, *B* Binocular, *C* Corrected, *U* Uncorrected, *D* Distance, *I* Intermediate, *N* Near, *P* Photopic, *Me* Mesopic, *WG* Without glare, *G* With glare, *All* All the CS standardized conditions were tested, *All*- All the CS standardized conditions were tested excepting the included, &: Joins different conditions

### EDoF criteria

Although four randomized clinical trials (RCT) were identified, [39, 43, 45, 46] none accomplished all the requirements described by the ANSI for evaluating the EDOF criteria. Table 5 summarizes the criteria for classifying an intraocular lens as an EDOF according to the ANSI standard. Three possible answers were covered in the table, “yes” and “not” when the criteria were or not accomplished, respectively, according to the reviewed paper, and “unclear” when not enough information was provided by the study to provide an answer. Only complete agreement was obtained for ANSI-1 and ANSI-4, indicating no inferiority at distance and better visual acuity at intermediate with the best distance correction in comparison to the standard monofocal. Controversy, but with a higher number of studies reporting no accomplishment of this criteria, was found for the ANSI-2 which describes that the depth of focus was not generally increased above 0.5 D for a visual acuity of 0.2 logMAR. Furthermore, no studies reported the accomplishment of the ANSI-3 criteria that was generally not reported or not accomplished according to two studies [3, 49]. Additional end-points that were not included in the standards are described in Table 5.

**Table 5:**
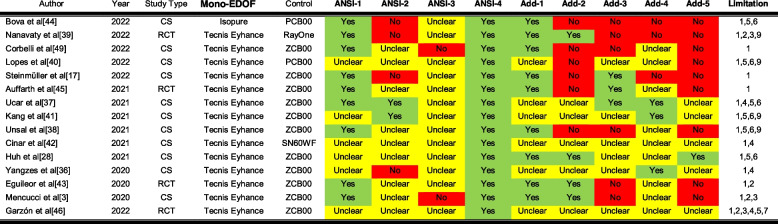
Criteria accomplished by the enhanced monofocals according to the ANSI standard definition of EDOF lenses and additional points

*RCT* Randomized clinical trial, *CS* Case series

ANSI-1: Statistical significance versus control for mean monocular visual acuity, with distance correction, at an intermediate distance of 66 cm

ANSI-2: An increase in depth of field is achieved in the monocular defocus curve with distance correction equal to or greater than 0.5 D for a visual acuity level of 0.2 logMAR

ANSI-3: The median monocular visual acuity, with distance correction, at an intermediate distance of 66 cm, is at least 0.2 logMAR

ANSI-4: Monocular acuity at distance, with correction at this distance, is statistically non-inferior to control using a non-inferiority margin of 0.1 logMAR

Add-1: Statistical significance versus control for mean binocular visual acuity, with distance correction, at an intermediate distance of 66 cm

Add-2: An increase in depth of field is achieved in the binocular defocus curve with distance correction equal to or greater than 0.5 D for a visual acuity level of 0.2 logMAR

Add-3: The mean monocular visual acuity, with distance correction, at an intermediate distance of 66 cm, is at least 0.2 logMAR

Add-4: Starting with CDVA, the monocular defocus range at 0.2 logMAR is ≥ 1.5 and < 2.5

Add-5: Starting with CDVA, the binocular defocus range at 0.2 logMAR is ≥ 2.0 and < 3.0

Study limitation

1.- Lower sample size than described by ANSI

2.- Preoperative corneal astigmatism greater than that described by ANSI (1.0 D)

3.- Corneal incisions applied for astigmatism correction

4.- The evaluation intermediate distance is not 66 cm indicated by ANSI

5.- The chart used for testing visual acuity and defocus range is not the standard ETDRS or it has not been reported

6.- The chart used for testing intermediate visual acuity is not the standard ETDRS (i.e. Jaeger, Snellen, etc.) or it has not been reported

7.- The descriptive statistics is computed for a notation different of logMAR

8.- Control was not a standard monofocal

9.- Defocus curves were not measured with best distance correction

Field color codes: Red (not accomplished), Green (accomplished), Yellow (unreported or unclear)

Bova et al. [44], Nanavaty et al. [39], Corbelli et al. [49], Lopes et al. [40], Steinmüller et al. [17], Auffarth et al. [45], Ucar et al. [37], Kang et al. [41], Unsal et al. [38], Cinar et al. [42], Huh et al. [28], Yangzes et al. [36], Eguileor et al. [43], Mencucci et al. [3], Garzón et al. [46]

## Discussion

In this scoping review, the results reported by laboratory and clinical evidence for enhanced monofocal IOLs have been explored. Two systematic reviews, 13 laboratory studies, 21 clinical studies, and two mixed studies were included. The systematic reviews only included evidence for the Tecnis Eyhance IOL, which was for both laboratory and clinical studies, the enhanced monofocal IOL with the highest volume of evidence to date. Laboratory studies have reported optical quality of other enhanced monofocal IOLs such as RayOne EMV [20, 23–25], IsoPure [20, 27], Zoe [20], Acunex Quantum [21], Lentis Quantum [22], and Vivinex Impress [27], but optical profiles have been only documented for Tecnis Eyhance [31, 32]. However, laboratory or clinical studies have not been conducted for other announced enhanced monofocal IOLs such as Evolux and Extend HP.

Regarding the laboratory evidence, the elevation changes for refractive EDoF lenses were not achieved for the Tecnis Eyhance [31, 32], and the optical quality studies that included both, enhanced monofocals and EDoF lenses, showed a higher depth of focus range with poorer distance optical quality for EDoF lenses in comparison to enhanced monofocals [25, 27]. These laboratory results were in agreement with clinical evidence of studies including enhanced monofocals and EDoF lenses, demonstrating a higher range of focus and better DCNVA for the latter [33–35, 49]. Interestingly, no significant differences were reported for visual acuities at intermediate vision [33–35]. These results suggest that enhanced monofocal IOLs might provide comparable visual acuity at intermediate vision in comparison to the EDoF and the most important differences might be found at near vision. However, further RCT comparing enhanced monofocals and EDoF lenses are required to conduct a future systematic review that provides evidence to answer this question.

Although enhanced monofocals have demonstrated superior intermediate vision in comparison to standard monofocals, the EDoF criteria checked in this scoping review evidence that the increase in the range of vision might not be enough to classify these lenses as EDoF lenses. According to the ANSI, for a lens to be classified as EDoF, it must to meet each and every one of the four criteria included in Table 5. Unfortunately, published studies rarely present the results of these four criteria, in many cases, because the authors chose to report the results binocularly rather than monocularly or the mean rather than the median, as described by the ANSI. For this reason, although not described by ANSI, we extended the comparison using five additional criteria that would help to better understand lens performance when ANSI criteria have not been reported (Table 5).

### Strengths and limitations

The objective of this scoping review was to map the laboratory and clinical evidence of popularly known enhanced monofocal IOLs. A systematic approach was conducted following the recommendations for this review type, which is a major strength compared to non-systematic approaches such as narrative reviews. On the other hand, the wide scope does not allow the evaluation of effectiveness as in systematic reviews and meta-analysis, but recent meta-analyses have been already published answering to the efficacy question [6, 7]. These meta-analysis also included case series, with a higher number of references in the Wan et al. [6] meta-analysis.

Our scoping review also included three cases series that were not included in the previous ones, [17, 37, 44] but the results provided in these three references were in agreement with the conclusions reported by Wan et al. [6]. This scoping review also included a comparison study for IsoPure [44] IOL in contrast to previous systematic reviews that only included Tecnis Eyhance. It is also important to note that Wan et al. [6] incorrectly included a study cited as Alió 2021 in some endpoints of their meta-analysis, whereas Tecnis Eyhance was not included in Alió’s study. A major strength of this scoping review in comparison with the previously published meta-analysis is the inclusion of lenses beyond Tecnis Eyhance, the inclusion of studies comparing results with EDoF lenses, and the exploration of the accomplishment of the EDoF criteria for ANSI standards. Unfortunately, a major limitation is that there is no standard definition of enhanced monofocal IOLs; therefore, the lenses included in the context are those that, according to the reviewers, might be qualified as enhanced monofocals. The findings summarized in this scoping review could help the reader to compare the results between the reviewed IOLs, but until future standards consider the inclusion of a new category and define the criteria for this category, all these lenses can be considered as monofocal lenses, considering that associating the term “enhanced” to the lenses might be arbitrary because of the lack of standard criteria.

## Conclusion

This review identified 39 studies divided into two systematic reviews, 13 laboratory studies, 21 clinical studies, and two mixed studies. Up to 11 laboratory studies and 17 clinical studies included optical, surface profile, and clinical results for Tecnis Eyhance, which means that approximately 74% of the current evidence of enhanced monofocals comes from this particular IOL. Conversely, although laboratory evidence can be found from other enhanced monofocal IOLs, only IsoPure and Xact IOLs provided clinical evidence in four studies, three case series without a comparison group, and only one case series comparing a standard monofocal IOL with the IsoPure IOL. Sufficient clinical evidence in comparison to standard monofocals has been provided by Tecnis Eyhance for the assessment of its efficacy in two published meta-analyses; however, for the remaining IOLs, clinical evidence is still very scarce. All the studies for which the ANSI criteria for EDoF lens classification could be checked corresponded to Tecnis Eyhance, except one study for IsoPure. For the four criteria that should be met to qualify these IOLs as EDoFs, ANSI-2 was not generally met, and no studies supported that the ANSI-3 criteria were met. In comparison with EDoF lenses, evidence is scarce, even for Tecnis Eyhance. Only four studies compared enhanced monofocals with EdoF lenses, particularly for Tecnis Eyhance, and only one study included three groups with monofocal, enhanced monofocal, and EDoF lenses. Tecnis Eyhance was positioned in this study with an increase in depth of focus between the standard monofocal and EDoF. Because of the lack of a standard classification for these lenses and because the ANSI standards were not fully met, these lenses should be better classified as monofocal until the international standards consider a new classification between monofocal and EDoF lenses.

## Supplementary Information

**Additional file 1.**

**Additional file 2.**

## Data Availability

Data extracted is provided in Tables and Supplemental File.
