# Supplementary material for: Positioning of enhanced monofocal intraocular lenses between conventional monofocal and extended depth of focus lenses: a scoping review
Source: BMC Ophthalmol. 2023 Mar 14;23:101. doi: 10.1186/s12886-023-02844-1 (PMC10015679; doi:10.1186/s12886-023-02844-1)
Supplement: Supplementary file 1 — Additional file 1. [file 12886_2023_2844_MOESM1_ESM.pdf]

Appendices

Appendix I: Search strategy

Table A.1. MEDLINE (Pubmed) search
Date searched: October 2, 2022

Search	Query	Entries
#1 Population	((Models, Theoretical*[mh] OR Vitro[tiab] OR Laboratory [tiab] OR "Optical performance"[tiab] OR Optics and Photonics*[mh]) OR (implantation[tiab] OR "Lens Implantation, Intraocular"[mh] OR cataract*[tiab] OR cataract[mh]))	4,018,088
#2 Concept	((profil*[ti] OR chromatic[tiab] OR through-focus[tiab] OR mtf[tiab] OR "modulation transfer function"[tiab] OR "simulated visual acuity"[tiab]) OR ("defocus curve*"[tiab] OR "spectacle dependence"[tiab] OR "Patient Reported Outcome Measures"[Mesh:no exp] OR (Glare[tiab] OR Halo[tiab] OR Starbust[tiab] OR dysphotopsia[tiab]) OR "patient satisfaction"[Mesh] OR "contrast sensitivity"[tiab] OR "contrast sensitivity"[Mesh:no exp] OR "visual acuity"[Mesh:no exp] OR "visual acuit*"[tiab] OR satisfaction[tiab] OR "spectacle independence"[tiab]))	560,791
#3 Context	(2020:2022[pdat] AND (((intraocular lens*[ti] OR "Lenses, Intraocular"[mh] OR iol[ti]) AND (enhanced monofocal*[tiab] OR "enhanced intermediate "[tiab] OR "new monofocal"[tiab])) OR (icb00[tiab] OR isopure[tiab] OR mono-EDOF[tiab] OR zoe[tiab] OR Eyhance[tiab] OR "vivinex impress"[tiab] OR xact[tiab] OR evolux[tiab] OR quantum[tiab] OR acunex[tiab])))	33,046
#4	#1 AND #2 AND #3	56

Table A.2. EMBASE (Elsevier)
Date searched: October 2, 2022

Search	Query	Entries
#1 Population	(('theoretical model'/exp OR Vitro:ti,ab,kw OR Laboratory:ti,ab,kw OR 'Optical performance':ti,ab,kw OR 'optics'/exp) OR (implantation:ti,ab,kw OR 'lens implantation'/exp OR cataract*:ti,ab,kw OR cataract/exp))	3,086,327
#2 Concept	((profil*:ti OR chromatic:ti,ab,kw OR through-focus:ti,ab,kw OR mtf:ti,ab,kw OR 'modulation transfer function':ti,ab,kw OR 'simulated visual acuity':ti,ab,kw) OR ('defocus curve*':ti,ab,kw OR 'spectacle dependence':ti,ab,kw OR 'patient-reported outcome'/de OR (Glare:ti,ab,kw OR Halo:ti,ab,kw OR Starbust:ti,ab,kw OR dysphotopsia:ti,ab,kw) OR 'patient satisfaction'/exp OR 'contrast sensitivity':ti,ab,kw OR 'contrast sensitivity'/de OR 'visual acuity'/de OR 'visual acuit*':ti,ab,kw OR satisfaction:ti,ab,kw OR 'spectacle independence':ti,ab,kw))	772,927
#3 Context	([2020-2022]/py AND ((('intraocular lens*':ti OR 'lens implant'/exp OR iol:ti) AND ('enhanced monofocal*':ti,ab,kw OR 'enhanced intermediate':ti,ab,kw OR 'new monofocal':ti,ab,kw)) OR (icb00:ti,ab,kw OR isopure:ti,ab,kw OR mono-EDOF:ti,ab,kw OR zoe:ti,ab,kw OR Eyhance:ti,ab,kw OR 'vivinex impress':ti,ab,kw OR xact:ti,ab,kw OR evolux:ti,ab,kw OR quantum:ti,ab,kw OR acunex:ti,ab,kw)))	29,265
#4	#1 AND #2 AND #3	46

Table A.3. WEB OF SCIENCE (Isi web of knowledge)
Date searched: October 2, 2022 (Filtered from 2020 to 2022)

Search	Query	Entries
#1 Population	TS=((Vitro OR Laboratory OR 'Optical performance' OR implantation OR 'lens implantation' OR cataract*))	1,460,929
#2 Concept	TS=(profil* OR chromatic OR through-focus OR mtf OR 'modulation transfer function' OR 'simulated visual acuity' OR 'defocus curve*' OR 'spectacle dependence' OR 'patient-reported outcome'OR Glare OR Halo OR Starbust OR dysphotopsia OR 'patient satisfaction' OR 'contrast sensitivity' OR 'contrast sensitivity' OR 'visual acuity' OR 'visual acuit*' OR 'spectacle independence')	502,492
#3 Context	TS=(('intraocular lens') AND ('enhanced monofocal*' OR 'enhanced intermediate' OR 'new monofocal' OR icb00 OR isopure OR mono-EDOF OR zoe OR Eyhance OR 'vivinex impress' OR xact OR evolux OR quantum OR acunex))	100
#4	#1 AND #2 AND #3	74
