# Supplementary material for: Positioning of enhanced monofocal intraocular lenses between conventional monofocal and extended depth of focus lenses: a scoping review
Source: BMC Ophthalmol. 2023 Mar 14;23:101. doi: 10.1186/s12886-023-02844-1 (PMC10015679; doi:10.1186/s12886-023-02844-1)
Supplement: Supplementary file 2 — Additional file 2. [file 12886_2023_2844_MOESM2_ESM.pdf]

Table B.1. Outcomes from data extraction.

STUDY INFO	Identifier	Authors	Title	Journal	Year
	1	rán-Diego, Laura Rico-del-V	Visual and opt	Graefe's Archi	2022
	2	lessandro Bova, Stefano Vit	Clinical and Ab	Journal of Oph	2022
	3	Sean Gallagher, Steven Bo	anced Monofo	of Refractive S	2022
	4	enzo Iuliano, Francesco Ban	lar lenses: mo	aract and Refra	2022
	5	odrigues Barros, João Nobre	implantation c	ournal of Opht	2021
	6	David Rua Amaro, Eckart Be	Analysis of hig	European Jour	2022
	7	anakiraman, Beth Jackson, A	th enhanced ir	aract and Refra	2021
	8	Fikret Ucar, Servet Cetinkay	th of Focus to	hensive Clinica	2021
	9	ok Young Kim, Kyu-Yeon Hw	nplantation of	nal of Ophthalm	2021
	10	Ugur Unsal, Huri Sabur	standard monc	tional Ophthal	2021
	11	Erbakan, Berna Yuce, Fatih	es with a new	tional Ophthal	2021
	12	Seul Ki Yang, Young Choi, Hy	nd new mono	IC Ophthalmolo	2021
	13	eha Kamble, Sartaj Grewal, S	lens with the n	urnal of Ophth	2020
	14	art, N, Martínez Alday, C, Sa	ar monofocal v	ciedad Español	2020
	15	mo, Daniel Venturi, Robertc	a new monofo	aract and Refra	2020
	16	ok Chung, So Young Park, H	ntermediate fu	aract and Refra	2022
	17	Veindler, Tadas Naujokaitis,	d Monofocal IC	of Refractive S	2021
	18	n, Yisang Yoon, Tae-im Kim,	XR00) and a N	Journal of Oph	2021
	19	Belén Elvira-Giner, María Ri	pth of Focus IC	of Refractive S	2021
	20	Huri Sabur, Ugur Unsal	Visual outcom	European Jour	2022
	21	Pavel Stodulka, Martin Slova	mial Extended	urnal of Ophtha	2021

Volume	Pages	IOLModel	StudyType	omparison_wi	Timeline	Consecutive
	1-9	Eyhance_ICB0(	Randomized	Tecnis_ZCB00	Prospective	NA
4119698	7	IsoPure	Case Series	Tecnis PCB00	Retrospective	yes
38	10-20	Eyhance_ICB0(	Randomized	yOne_Monofo	Prospective	yes
48	67-74	Eyhance_ICB0(	Case Series	CB00 & Symfor	Prospective	yes
32	229-234	Eyhance_ICB0(	Case Series	Tecnis PCB00	Retrospective	yes
	1-10	Eyhance_ICB0(	Case Series	Tecnis_ZCB00	Prospective	yes
47	184-191	Eyhance_ICB0(	Randomized	Tecnis_ZCB00	Prospective	yes
3	2285–2291	Eyhance_ICB0(	Case Series	Tecnis_ZCB00	Retrospective	yes
35	112–119	Eyhance_ICB0(	Case Series	Tecnis_ZCB00	Retrospective	yes
41	273-282	Eyhance_ICB0(	Case Series	Tecnis_ZCB00	Retrospective	yes
41	491-498	Eyhance_ICB0(	Case Series	crySof SN60W	Retrospective	yes
21	365	Eyhance_ICB0(	Case Series	Tecnis_ZCB00	Retrospective	yes
68	3025	Eyhance_ICB0(	Case Series	Tecnis_ZCB00	Retrospective	yes
95	523-527	Eyhance_ICB0(	Randomized	Tecnis_ZCB00	Prospective	NA
46	378-387	Eyhance_ICB0(	Case Series	Tecnis_ZCB00	Prospective	yes
48	61-66	Eyhance_ICB0(	Case Series	Symfony ZXR0(	Prospective	yes
37	601-609	_Mono-EDOF_	Case Series	None	Prospective	yes
10	542-547	Eyhance_ICB0(	Case Series	Symfony ZXR0(	Retrospective	yes
37	595-600	_Mono-EDOF_	Case Series	None	Retrospective	yes
Ahead of print		Eyhance_ICB0(	Case Series	Vivity_DFT015	Prospective	yes
11	214-228	Isopure	Case Series	None	Retrospective	NA

RecruitLoss	POPULATION	Subjects	Eyes	neal_astigmat	Commorbilitie:	Age
NA		30	NA	1 to 1,5	No	75,87
NA		21	42	1	No	71,33
NA		25	50	0,75-2,50	No	72,79
No		25	50	0,75	No	72,7
No		30	60	1	No	71
Yes		15	30	0,75	No	65,8
Yes		67	134	1	No	69,3
NA		85	85	1	No	56,83
NA		111	111	1	No	65,23
NA		16	32	1	No	56,2
NA		63	63	1	No	61,3
NA		15	30	NA	No	69,6
No		71	71	1	No	65,43
No		15	30	1,5	No	72,2
No		20	40	0,75	No	72,31
NA		24	48	1,5	No	70,47
Yes		24	38	1	No	70,14
NA		102	102	1	No	65,2
No		25	50	1	No	67,66
No		18	36	1	No	54
NA		18	18	1	No	69,4

AgeSD	Cataract	Men%	Women%	AXL	AXLSD	Keratometry
5,97	Cat	22,4	77,6	23,3	1	44,61
7,91	Cat	NA	NA	23,74	0,33	43,83
8,04	Cat	NA	NA	24,07	1,56	43,59
4,3	Cat	NA	NA	23,72	0,63	43,76
5,4	Cat	47	53	23,1	0,8	44,25
9,45	Cat	44	56	23,84	1,75	NA
8,7	Cat	41,2	58,8	NA	NA	NA
8,54	Cat	51,7	48,3	23,45	0,95	NA
8,15	Cat	49	51	NA	NA	NA
7,8	Cat	62,5	37,5	NA	NA	NA
11,4	Cat	47,7	52,3	23,22	1,3	NA
7,3	Cat	33,3	66,7	24,13	1,38	43,78
6,7	Cat	47	53	23,59	0,4	43,98
7,7	Cat	53,3	46,7	23,71	0,74	NA
6,71	Cat	NA	NA	NA	NA	42,26
8,02	Cat	41,66	58,33	23,52	1,09	NA
9,76	Cat	43	57	NA	NA	NA
9,2	Cat	44	56	NA	NA	NA
7,15	Cat	48	52	23,74	1,25	43,53
6,8	Cat	44	56	23.46	1,25	43,01
6,9	Cat	38,9	61,1	23,25	0,79	NA

KeratometrySD	IOL_Power	IOL_PowerSD	CornealAst	CornealAstSD	ACD	ACDSD
1,42	21,96	2,23	NA	NA	3,04	0,36
NA	NA	NA	0,52	NA	3,03	0,33
1,71	NA	NA	1,05	0,57	3,15	1,56
NA	NA	NA	0,36	0,16	NA	NA
NA	22,6	1,1	NA	NA	NA	NA
NA	21,1	4,69	NA	NA	3,31	0,53
NA	NA	NA	NA	NA	NA	NA
NA	NA	NA	NA	NA	NA	NA
NA	NA	NA	NA	NA	NA	NA
NA	NA	NA	NA	NA	NA	NA
NA	NA	NA	-0,28	0,2	NA	NA
1,31	19,9	4,3	NA	NA	3,32	0,32
1,69	NA	NA	NA	NA	3,24	0,44
NA	21,52	1,96	0,84	0,42	NA	NA
NA	NA	NA	0,43	0,17	NA	NA
NA	NA	NA	0,66	0,44	NA	NA
NA	NA	NA	NA	NA	NA	NA
NA	NA	NA	NA	NA	NA	NA
NA	21,59	3,98	NA	NA	3,17	0,29
NA	NA	NA	0,5	0,23	NA	NA
NA	22,75	2,2	0,58	0,28	3,02	0,25

DevicePupil	PP	PPSD	MP	MPSD	CDVA	CDVASD
OPD-Scan III	3,21	0,56	4,32	0,84	0,19	0,15
Osiris	3,04	0,49	NA	NA	0,43	0,13
iTrace	NA	NA	4,31	0,51	0,47	0,42
NA	NA	NA	NA	NA	0,44	0,14
NA	NA	NA	NA	NA	NA	NA
NA	NA	NA	4,51	1,92	0,27	0,14
NA	NA	NA	NA	NA	NA	NA
NA	NA	NA	NA	NA	0,72	0,18
NA	NA	NA	NA	NA	0,33	0,31
NA	NA	NA	NA	NA	0,51	0,42
NA	NA	NA	NA	NA	0,53	0,2
NA	NA	NA	NA	NA	NA	NA
NA	NA	NA	NA	NA	0,55	0,55
Pentacam	2,4	0,31	NA	NA	NA	NA
NA	NA	NA	NA	NA	0,37	0,16
NA	NA	NA	NA	NA	0,22	0,25
PLR-3000	NA	NA	3,98	NA	NA	NA
NA	NA	NA	NA	NA	0,33	0,32
Pentacam	2,81	0,45	NA	NA	0,09	NA
NA	NA	NA	NA	NA	0,26	0,15
NA	NA	NA	NA	NA	0,26	0,13

SURGERY	Site	Surgeons	Phaco	CCI	CCI_Mer	Add_Inc
	NA	1	Standard	2,2	NA	NA
	Monfalcone Eye C	1	Standard	2,2	Superiorly	NA
	iversity Hospitals NHS trust, E	2	Standard	2,75	Sup-Temp	PCRIs
	of Ophthalmology of the San	1	Standard	2,4	Temporal	No
	Garcia de Orta Hospital, Alm	3	Standard	2,4	Steepest if >0,5	No
	phthalmology, Charité - Univ	1	Standard	2,2	Steepest	NA
	Multicentric	>1	Standard	2,2-3	NA	NA
	NA	1	Standard	2,4	NA	NA
	Kim's Eye Hospital, Seo	2	Standard	2,8	Steepest	NA
	NA	1	Standard	2,2	Steepest	NA
	Ekol Eye Hospit	1	Standard	2,2	NA	NA
	Korea University College	2	Standard	2,2-2,75	NA	NA
	NA	1	Standard	2,8	Temporal	NA
	lmology Department at Cruc	1	Standard	2,4	NA	NA
	Department of NEUROFARB	1	Standard	NA	Temporal	NA
	Ophthalmology, Asan Medic	1	Standard	2,2	Steepest	NA
	NA	2	Standard	NA	NA	NA
	Kim's Eye Hospital, Seoul, Re	1	Standard	2,8	Steepest	NA
	Oftalvist Clinic in Ali	NA	Femtosecond	2,2	Temporal	NA
	NA	1	Standard	2,2	Steepest	NA
	NA	NA	Standard	2,2	Steepest	NA

Nomogram	Capsulotomy	Target	TargetSD	Formula	Constant	SurgeryPlan
NA	NA	NA	NA	t Universal II fc	2.04	Emmetropia
NA	NA	NA	NA	NA	NA	NA
Donnenfield	NA	-0,13	0,12	SRK/T	119,3	Emmetropia
No	5,5	NA	NA	NA	NA	NA
No	5-5,5	NA	NA	arret Universal	NA	Emmetropia
NA	NA	NA	NA	versal II, Haigis	NA	Emmetropia
NA	5-5,5	NA	NA	NA	NA	Emmetropia
NA	NA	NA	NA	Holladay II	NA	Emmetropia
NA	5,2	-0,21	0,23	rrett Universa	NA	Emmetropia
NA	NA	NA	NA	NA	NA	NA
NA	NA	NA	NA	NA	NA	NA
NA	NA	-0,23	0,31	Haigis	)2, 0,210, and	Emmetropia
NA	NA	NA	NA	l Keratometry	NA	Emmetropia
NA	NA	NA	NA	SRK/T	119,3	Emmetropia
NA	5,5	NA	NA	laday 1 & Hoffe	NA	Emmetropia
NA	5	NA	NA	NA	NA	Emmetropia
NA	5	-0,13	0,11	Haigis	i4, 0,40, and 0	Emmetropia
NA	5,2	-0,32	0,2	rrett Universa	NA	NA
NA	5	NA	NA	rrett Universa	NA	Emmetropia
NA	5,5	NA	NA	NA	NA	NA
NA	NA	NA	NA	NA	NA	Emmetropia

Biometer	SAFETY	lossCDVA	cdva03	adverseEvents	ndYAG	EFFICACY
NA		NA	NA	None	NA	
NA		NA	NA	None	0	
laster 500 or A-Scan		NA	NA	NA	0	
OL Master 700		NA	NA	None	NA	
OL Master 700		NA	NA	Excluded	NA	
IOLMaster 700		NA	NA	6,67%	0	
NA		NA	NA	Yes	NA	
Nidek Biometry		NA	NA	NA	NA	
NA		NA	NA	NA	NA	
NA		NA	NA	NA	NA	
NA		NA	NA	Excluded	0	
OL Master 500		NA	NA	Excluded	NA	
OL Master 700		NA	NA	NA	NA	
OL Master 500		NA	NA	NA	NA	
OL Master 500		NA	NA	None	0	
OL Master 500		NA	NA	None	NA	
Master 500 or 700		NA	NA	NA	0	
Anterion		NA	NA	None	NA	
OL Master 700		NA	NA	None	NA	
IOLMaster 700		NA	NA	None	0	
NA		NA	NA	NA	NA	

FarDistance	InterDistance	NearDistance	MonoFollow	MonoUDVA	MonoUDVASD	MonoCDVA
4	NA	NA	1	0,16	0,24	0,02
4	66	NA	12	0,04	0,05	0,03
4	66	NA	6	0,04	0,09	-0,03
4	66	40	6	0,01	0,02	0
NA	66	NA	3	0,04	0,18	NA
4	66	40	3	-0,05	0,11	-0,15
4	66	NA	6	NA	NA	-0,02
5	70	40	6	0,07	0,05	0,05
4	66	40	3	0,11	0,1	0,04
4	66	40	3	0,04	0,07	0,03
4	60	40	3	0,05	0,13	0,02
4	66	40	1	0,06	0,1	-0,01
4	80	40	2weeks	0,1	0,13	0,02
4	66	NA	1	NA	NA	0,02
4	66	40	6	0,04	0,05	0,02
4	66	40	3	0,04	0,06	0,02
4	70	NA	6	0,08	0,15	0,07
4	66	40	3	0,11	0,1	0,03
NA	NA	NA	12	NA	NA	NA
4	66	40	3	0,05	0,08	0,02
4	66	40	6	0,03	0,13	-0,06

MonoCDVASD	MonoF2020%	MonoUIVA	MonoUIVASD	MonoDCIVA	MonoDCIVASD	MonoI2025%
0,05	NA	NA	NA	NA	NA	NA
0,05	NA	0,24	0,11	0,23	0,07	NA
0,08	NA	0,29	0,15	0,27	0,17	NA
0,02	NA	0,28	0,06	0,25	0,07	NA
NA	NA	0,21	0,12	NA	NA	NA
0,09	NA	0,07	0,12	0,08	0,11	NA
0,12	NA	0,16	0,23	0,19	0,23	NA
0,03	NA	0,08	0,07	0,07	0,06	NA
0,06	NA	0,24	0,1	NA	NA	NA
0,05	NA	0,24	0,16	0,28	0,31	NA
0,02	NA	0,31	0,16	0,28	0,12	NA
0,09	NA	NA	NA	NA	NA	NA
0,05	NA	0,11	0,13	NA	NA	NA
0,07	NA	NA	NA	0,26	0,13	NA
0,04	NA	0,28	0,11	0,27	0,11	NA
0,04	NA	0,16	0,12	NA	NA	NA
0,08	NA	0,18	0,16	0,25	0,14	NA
0,05	NA	0,26	0,09	NA	NA	NA
NA	92	NA	NA	NA	NA	NA
0,04	NA	0,17	0,09	0,17	0,08	NA
0,04	100	0,26	0,15	0,27	0,13	16,7

MonoUNVA	MonoUNVASD	MonoDCNVA	MonoDCNVASI	MonoN2025%	BinoFollow	BinoUDVA
NA	NA	NA	NA	NA	NA	NA
NA	NA	NA	NA	NA	12	0,03
NA	NA	NA	NA	NA	6	-0,05
0,32	0,04	0,32	0,04	NA	6	0,01
NA	NA	NA	NA	NA	3	0,04
0,26	0,11	0,25	0,21	NA	3	-0,12
NA	NA	NA	NA	NA	6	0,03
0,87	0,63	0,44	0,18	NA	NA	NA
0,46	0,14	NA	NA	NA	NA	NA
NA	NA	0,55	0,45	NA	3	NA
0,47	0,21	0,46	0,17	NA	NA	NA
NA	NA	NA	NA	NA	1	NA
0,43	0,13	NA	NA	NA	NA	NA
NA	NA	NA	NA	NA	1	NA
0,46	0,13	0,44	0,13	NA	6	0,03
0,51	0,27	NA	NA	NA	3	0,01
NA	NA	NA	NA	NA	6	-0,02
0,42	0,11	NA	NA	NA	NA	NA
NA	NA	NA	NA	NA	NA	NA
0,5	0,09	0,5	0,08	NA	3	0,03
NA	NA	NA	NA	NA	5	-0,02

BinoUDVASD	BinoF2020%	BinoCDVA	BinoCDVASD	BinoUIVA	BinoUIVASD	BinoI2025%
NA	NA	NA	NA	NA	NA	NA
0,04	NA	0,01	0,03	0,22	0,06	NA
0,09	72,7	-0,08	0,07	0,13	0,1	NA
0,02	92	0	0,02	0,04	0,04	88
0,12	NA	0,03	0,09	0,17	0,1	NA
0,1	NA	-0,2	0,08	-0,04	0,06	NA
0,12	NA	-0,06	0,09	0,07	0,12	NA
NA	NA	NA	NA	NA	NA	NA
NA	NA	NA	NA	NA	NA	NA
NA	NA	NA	NA	0,18	NA	NA
NA	NA	NA	NA	NA	NA	NA
NA	NA	-0,04	0,09	0,03	0,06	NA
NA	NA	NA	NA	NA	NA	NA
NA	NA	-0,05	0,07	NA	NA	NA
0,05	64	0,01	0,04	0,16	0,1	50
0,03	NA	0,01	0,03	0,08	0,11	NA
0,09	28	-0,09	0,09	0,09	0,12	NA
NA	NA	NA	NA	NA	NA	NA
NA	NA	NA	NA	NA	NA	NA
0,04	NA	0,02	0,04	0,12	0,05	NA
0,13	83,3	-0,09	0,06	0,2	0,14	35,3

BinoDCIVA	BinoDCIVASD	BinoUNVA	BinoUNVASD	BinoN2025%	BinoDCNVA	BinoDCNVASD
NA	NA	NA	NA	NA	NA	NA
0,21	0,07	NA	NA	NA	NA	NA
0,14	0,08	NA	NA	81,8	NA	NA
0,07	0,06	0,28	0,05	0	0,31	0,03
NA	NA	NA	NA	NA	NA	NA
0,03	0,13	0,08	0,1	NA	0,19	0,16
0,09	0,11	NA	NA	NA	NA	NA
NA	NA	NA	NA	NA	NA	NA
NA	NA	NA	NA	NA	NA	NA
0,2	NA	NA	NA	NA	NA	NA
NA	NA	NA	NA	NA	NA	NA
0,01	0,04	0,09	0,14	NA	0,09	0,15
NA	NA	NA	NA	NA	NA	NA
-0,16	0,12	NA	NA	NA	NA	NA
0,15	0,08	0,33	0,05	0	0,32	0,04
NA	NA	0,33	0,15	NA	NA	NA
0,13	0,11	NA	NA	NA	NA	NA
NA	NA	NA	NA	NA	NA	NA
NA	NA	NA	NA	NA	NA	NA
0,12	0,04	0,44	0,08	NA	0,43	0,06
0,2	0,11	NA	NA	NA	NA	NA

ResidualSE	ResidualSESD	ResidualAst	ResidualAstSD	Accuracy05	Accuracy10	ETDRS
-0,13	NA	NA	NA	NA	NA	Yes
NA	NA	NA	NA	NA	NA	Yes
-0,02	0,41	-0,63	0,44	81,81	97,72	Yes
NA	NA	NA	NA	100	100	Yes
NA	NA	NA	NA	87	NA	NA
-0,29	0,48	0,46	0.49	NA	NA	Yes
-0,25	0,05	0,5	0,04	74,6	94	Yes
0,43	0,12	-0,29	0,14	NA	NA	NA
-0,22	0,39	NA	NA	93	NA	NA
-0,36	0,52	-0,34	0,19	62,5	84	No
NA	NA	NA	NA	NA	NA	Yes
-0,16	0,37	NA	NA	NA	NA	NA
-0,017	0,33	-0,08	0,39	NA	NA	Yes
-0,3	0,44	NA	NA	NA	NA	Yes
-0,33	0,49	NA	NA	62,5	92	Yes
-0,11	0,23	0,41	0,35	NA	NA	NA
-0,25	0,4	NA	NA	NA	NA	Yes
-0,22	0,4	NA	NA	92	NA	Yes
NA	NA	-0,44	0,36	95	100	No
-0,06	0,23	0,26	0,22	100	100	No
-0,16	0,46	-0,36	0,45	88,9	94,5	Yes

Luminance	DEFOCUS CURV	mFollowupDC	mDCDC	biasPrev	mDC20	mDC15
85		NA	NA	NA	NA	NA
NA		1	No	NA	NA	NA
85		6	yes	NA	NA	0,27
85		NA	NA	NA	NA	NA
NA		NA	NA	NA	NA	NA
85		3	yes	NA	NA	0,28
85		NA	NA	NA	NA	NA
NA		6	yes	NA	0,53	0,40
NA		3	NA	NA	NA	NA
85		NA	NA	NA	NA	NA
NA		NA	NA	NA	NA	NA
NA		1	yes	NA	NA	NA
167		2 weeks	yes	NA	NA	NA
85		NA	NA	NA	NA	NA
85		NA	NA	NA	NA	NA
NA		3	yes	NA	NA	NA
NA		6	yes	NA	0,59	0,42
NA		3	NA	NA	NA	NA
NA		12	yes	NA	0,50	0,33
85		NA	NA	NA	NA	NA
NA		5	yes	NA	NA	0,51

mDC10	mDC05	mDC00	mDC-05	mDC-10	mDC-15	mDC-20
NA	NA	NA	NA	NA	NA	NA
0,25	0,13	0,00	0,09	0,18	0,27	0,44
0,16	0,03	0,01	0,00	0,08	0,24	0,39
NA	NA	NA	NA	NA	NA	NA
NA	NA	NA	NA	NA	NA	NA
0,11	-0,05	-0,15	-0,05	0,06	0,21	0,37
NA	NA	NA	NA	NA	NA	NA
0,18	0,10	0,02	0,09	0,14	0,17	0,25
0,11	0,06	0,04	0,03	0,08	0,18	0,26
NA	NA	NA	NA	NA	NA	NA
NA	NA	NA	NA	NA	NA	NA
NA	NA	NA	NA	NA	NA	NA
0,27	0,11	0,02	0,04	0,1	0,2	0,34
NA	NA	NA	NA	NA	NA	NA
NA	NA	NA	NA	NA	NA	NA
NA	0,15	0,07	0,11	0,15	0,21	0,27
0,21	0,07	-0,03	0,04	0,16	0,33	0,47
0,34	0,22	0,11	0,16	0,21	0,26	0,37
0,19	0,06	0,02	0,05	0,16	0,29	0,44
NA	NA	NA	NA	NA	NA	NA
0,34	0,17	-0,01	0,08	0,18	0,31	0,44

mDC-25	mDC-30	mDC-35	mDC-40	bFollowupDC	bDCDC	bDC20
NA	NA	NA	NA	NA	NA	NA
NA	NA	NA	NA	12	No	NA
0,50	0,65	NA	NA	6	Yes	NA
NA	NA	NA	NA	6	Yes	NA
NA	NA	NA	NA	3	NA	NA
0,54	0,62	0,71	0,81	3	Yes	NA
NA	NA	NA	NA	1	Yes	NA
0,33	NA	NA	NA	NA	NA	NA
0,33	NA	NA	NA	NA	NA	NA
NA	NA	NA	NA	3	NA	NA
NA	NA	NA	NA	NA	NA	NA
NA	NA	NA	NA	1	Yes	NA
0,46	0,57	0,71	0,82	NA	NA	NA
NA	NA	NA	NA	1	Yes	NA
NA	NA	NA	NA	6	Yes	NA
0,40	0,52	0,71	0,87	3	Yes	NA
0,65	NA	NA	NA	6	Yes	NA
0,43	0,49	NA	NA	NA	NA	NA
0,60	0,84	1,16	1,84	NA	NA	NA
NA	NA	NA	NA	3	NA	NA
0,63	NA	NA	NA	5	Yes	NA

bDC15	bDC10	bDC05	bDC00	bDC-05	bDC-10	bDC-15
NA	NA	NA	NA	NA	NA	NA
NA	0,25074627	0,11343284	2,78E-17	0,07761194	0,15970149	0,22537313
0,16	0,06	-0,02	-0,02	-0,04	0,02	0,13
NA	0,12	0,05	0,01	0,05	0,09	0,19
0,50	0,30	0,20	0,01	0,11	0,21	0,32
0,22	0,07	-0,10	-0,19	-0,11	0,00	0,12
NA	0,19	0,06	-0,05	0,00	0,10	0,22
NA	NA	NA	NA	NA	NA	NA
NA	NA	NA	NA	NA	NA	NA
NA	0,12	0,05	-0,01	0,06	0,10	0,25
NA	NA	NA	NA	NA	NA	NA
NA	0,04	-0,02	-0,04	-0,01	-0,01	0,03
NA	NA	NA	NA	NA	NA	NA
NA	NA	0,04	-0,05	0,00	0,04	0,16
NA	0,08	0,04	0,01	0,05	0,09	0,20
NA	NA	0,15	0,02	0,04	0,06	0,09
0,30	0,13	0,01	-0,09	-0,03	0,09	0,25
NA	NA	NA	NA	NA	NA	NA
NA	NA	NA	NA	NA	NA	NA
NA	0,121875	0,05	0,0125	0,071875	0,121875	0,23125
0,39	0,23	0,09	-0,04	0,01	0,09	0,21

bDC-20	bDC-25	bDC-30	bDC-35	bDC-40	TRAST SENSITI	testCS
NA	NA	NA	NA	NA		CSV-1000
0,39402985	NA	NA	NA	NA		CSO
0,25	0,38	0,50	NA	NA		NA
0,31	0,42	NA	NA	NA		FACT
0,52	0,72	0,82	0,93	NA		NA
0,29	0,43	0,54	0,65	0,80		FACT
0,33	NA	NA	NA	NA		M&S
NA	NA	NA	NA	NA		NA
NA	NA	NA	NA	NA		NA
0,32	0,39	NA	NA	NA		NA
NA	NA	NA	NA	NA		NA
0,09	0,18	0,28	NA	NA		NA
NA	NA	NA	NA	NA		NA
0,32	0,43	NA	NA	NA		NA
0,29	0,39	NA	NA	NA		FACT
0,15	0,23	0,30	0,41	0,53		NA
0,38	0,54	NA	NA	NA		CSV-1000
NA	NA	NA	NA	NA		NA
NA	NA	NA	NA	NA		NA
0,315625	0,403125	NA	NA	NA		FACT
0,38	0,51	NA	NA	NA		CSV-1000

ceilingFloor	mFollowupCS	mDCCS	m3PWG	m6PWG	m12PWG	m18PWG
NA	1	Yes	1,57	1,62	1,15	0,66
NA	NA	NA	NA	NA	NA	NA
NA	NA	NA	NA	NA	NA	NA
NA	NA	NA	NA	NA	NA	NA
NA	NA	NA	NA	NA	NA	NA
NA	3	Yes	1,98	2,06	1,64	1,19
NA	6	Yes	NA	NA	NA	NA
NA	NA	NA	NA	NA	NA	NA
NA	NA	NA	NA	NA	NA	NA
NA	NA	NA	NA	NA	NA	NA
NA	NA	NA	NA	NA	NA	NA
NA	NA	NA	NA	NA	NA	NA
NA	NA	NA	NA	NA	NA	NA
NA	NA	NA	NA	NA	NA	NA
NA	NA	NA	NA	NA	NA	NA
NA	NA	NA	NA	NA	NA	NA
NA	6	Yes	1,63	1,85	1,53	1,16
NA	NA	NA	NA	NA	NA	NA
NA	NA	NA	NA	NA	NA	NA
NA	NA	NA	NA	NA	NA	NA
NA	1	NA	1,62	1,83	1,46	0,97

m15MWG	m3MWG	m6MWG	m12MWG	m3PG	m6PG	m12PG
NA	NA	NA	NA	NA	NA	NA
NA	NA	NA	NA	NA	NA	NA
NA	NA	NA	NA	NA	NA	NA
NA	NA	NA	NA	NA	NA	NA
NA	NA	NA	NA	NA	NA	NA
1,86	1,93	1,89	1,48	NA	NA	NA
2,03	1,99	1,72	1,06	1,22	1,48	1,42
NA	NA	NA	NA	NA	NA	NA
NA	NA	NA	NA	NA	NA	NA
NA	NA	NA	NA	NA	NA	NA
NA	NA	NA	NA	NA	NA	NA
NA	NA	NA	NA	NA	NA	NA
NA	NA	NA	NA	NA	NA	NA
NA	NA	NA	NA	NA	NA	NA
NA	NA	NA	NA	NA	NA	NA
NA	NA	NA	NA	NA	NA	NA
NA	1,52	1,43	0,84	1,64	1,87	1,5
NA	NA	NA	NA	NA	NA	NA
NA	NA	NA	NA	NA	NA	NA
NA	NA	NA	NA	NA	NA	NA
NA	1,45	1,42	0,98	NA	NA	NA

m18PG	m15MG	m3MG	m6MG	m12MG	bFollowupCS	bDCCS
NA	NA	NA	NA	NA	NA	NA
NA	NA	NA	NA	NA	12	No
NA	NA	NA	NA	NA	NA	NA
NA	NA	NA	NA	NA	6	NA
NA	NA	NA	NA	NA	NA	NA
NA	1,8	1,89	1,89	1,23	3	Yes
0,92	1,2	1,39	1,26	0,41	NA	NA
NA	NA	NA	NA	NA	NA	NA
NA	NA	NA	NA	NA	NA	NA
NA	NA	NA	NA	NA	NA	NA
NA	NA	NA	NA	NA	NA	NA
NA	NA	NA	NA	NA	NA	NA
NA	NA	NA	NA	NA	NA	NA
NA	NA	NA	NA	NA	NA	NA
NA	NA	NA	NA	NA	6	NA
NA	NA	NA	NA	NA	NA	NA
1,05	NA	1,44	1,31	0,86	6	NA
NA	NA	NA	NA	NA	NA	NA
NA	NA	NA	NA	NA	NA	NA
NA	NA	NA	NA	NA	3	NA
NA	NA	NA	NA	NA	5	NA

b3PWG	b6PWG	b12PWG	b18PWG	b15MWG	b3MWG	b6MWG
NA	NA	NA	NA	NA	NA	NA
1,78	1,85	1,44	1,22	NA	NA	NA
NA	NA	NA	NA	NA	NA	NA
2,03	2,08	1,77	1,56	NA	NA	NA
NA	NA	NA	NA	NA	NA	NA
2,01	2,08	1,78	1,3	1,91	2,01	2
NA	NA	NA	NA	NA	NA	NA
NA	NA	NA	NA	NA	NA	NA
NA	NA	NA	NA	NA	NA	NA
NA	NA	NA	NA	NA	NA	NA
NA	NA	NA	NA	NA	NA	NA
NA	NA	NA	NA	NA	NA	NA
NA	NA	NA	NA	NA	NA	NA
NA	NA	NA	NA	NA	NA	NA
1,92	2,04	1,72	1,5	NA	NA	NA
NA	NA	NA	NA	NA	NA	NA
1,73	1,93	1,56	1,12	NA	1,53	1,5
NA	NA	NA	NA	NA	NA	NA
NA	NA	NA	NA	NA	NA	NA
NA	NA	NA	NA	NA	NA	NA
1,61	1,84	1,46	0,94	NA	1,54	1,4

b12MWG	b3PG	b6PG	b12PG	b18PG	b15MG	b3MG
NA	NA	NA	NA	NA	NA	NA
NA	NA	NA	NA	NA	NA	NA
NA	NA	NA	NA	NA	NA	NA
NA	NA	NA	NA	NA	NA	NA
NA	NA	NA	NA	NA	NA	NA
1,4	NA	NA	NA	NA	1,83	1,93
NA	NA	NA	NA	NA	NA	NA
NA	NA	NA	NA	NA	NA	NA
NA	NA	NA	NA	NA	NA	NA
NA	NA	NA	NA	NA	NA	NA
NA	NA	NA	NA	NA	NA	NA
NA	NA	NA	NA	NA	NA	NA
NA	NA	NA	NA	NA	NA	NA
NA	NA	NA	NA	NA	NA	NA
NA	NA	NA	NA	NA	NA	NA
NA	NA	NA	NA	NA	NA	NA
1	1,8	1,98	1,62	1,18	NA	1,56
NA	NA	NA	NA	NA	NA	NA
NA	NA	NA	NA	NA	NA	NA
NA	NA	NA	NA	NA	NA	NA
0,97	NA	NA	NA	NA	NA	NA

b6MG	b12MG	REPORTED OL	followupPROs	farSI	interSI	nearSI
NA	NA		NA	NA	NA	NA
NA	NA		NA	NA	NA	NA
NA	NA		6	NA	NA	NA
NA	NA		NA	NA	80	12
NA	NA		3	NA	NA	NA
1,87	1,26		3	NA	NA	NA
NA	NA		6	NA	NA	NA
NA	NA		NA	NA	NA	NA
NA	NA		NA	NA	NA	NA
NA	NA		NA	97	84	6
NA	NA		NA	95.2	90.4	55.5
NA	NA		1	NA	NA	NA
NA	NA		NA	NA	NA	NA
NA	NA		NA	NA	NA	NA
NA	NA		6	100	80	5
NA	NA		3	NA	NA	16
1,43	0,81		6	NA	NA	NA
NA	NA		NA	NA	NA	NA
NA	NA		NA	NA	NA	NA
NA	NA		3	NA	NA	8,3
NA	NA		NA	NA	NA	NA

satisfied	dissatisfied	halosPP	glarePP	starburstPP	halosPD	glarePD
NA	NA	NA	NA	NA	NA	NA
NA	NA	NA	NA	NA	NA	NA
NA	NA	0	0	NA	NA	NA
NA	NA	NA	NA	NA	NA	NA
100	0	15	16	NA	NA	NA
NA	NA	14,29	14,29	NA	NA	NA
NA	NA	NA	NA	NA	20	20
NA	NA	NA	NA	NA	NA	NA
NA	NA	NA	NA	NA	NA	NA
NA	NA	NA	NA	NA	3	3
NA	NA	NA	NA	NA	NA	NA
NA	NA	7	13	0	NA	NA
NA	NA	NA	NA	NA	NA	NA
NA	NA	NA	NA	NA	NA	NA
NA	NA	NA	NA	NA	NA	NA
83	0	0	NA	NA	7	15
NA	NA	54,5	59,1	14	NA	NA
NA	NA	NA	NA	NA	NA	NA
NA	NA	NA	NA	NA	NA	NA
80,5	NA	NA	NA	NA	0	0
NA	NA	NA	NA	NA	NA	NA

starburstPD	generalPD	recommended	Statistics
NA	NA	NA	Yes
NA	NA	NA	Yes
NA	NA	NA	Yes
NA	NA	NA	Yes
NA	NA	NA	No
NA	NA	NA	Yes
20	NA	NA	Yes
NA	NA	NA	No
NA	NA	NA	Yes
3	NA	NA	Yes
NA	NA	NA	Yes
NA	NA	NA	Yes
NA	NA	NA	Yes
NA	NA	NA	Yes
NA	NA	NA	Yes
NA	NA	92	Yes
NA	NA	NA	Yes
NA	NA	NA	Yes
NA	NA	NA	Yes
NA	NA	NA	Yes
NA	NA	NA	Yes
